# Spatial analysis of the prevalence of abdominal obesity in middle-aged and older adult people in China: exploring the relationship with meteorological factors based on gender differences

**DOI:** 10.3389/fpubh.2024.1426295

**Published:** 2024-07-19

**Authors:** Chaohui Yin, Jinlong Yan, Junqi Wang, Tianyi Wang, Hangyu Li, Yuan Wang, Haifeng Wang, Shixing Feng, Yafeng Liang

**Affiliations:** ^1^School of Resources and Environment, Henan Agricultural University, Zhengzhou, Henan, China; ^2^Department of Geography and Spatial Information Techniques, Ningbo University, Ningbo, China; ^3^Dongzhimen Hospital, Beijing University of Chinese Medicine, Beijing, China; ^4^School of Management, Beijing University of Chinese Medicine, Beijing, China; ^5^School of Life Sciences, Beijing University of Chinese Medicine, Beijing, China; ^6^College of Acu-moxibustion and Massage, Shaanxi University of Chinese Medicine, Xianyang, China; ^7^Xiangyang Central Hospital, Affiliated Hospital of Hubei University of Arts and Science, Xianyang, China; ^8^School of Chinese Materia Medica, Beijing University of Chinese Medicine, Xianyang, China; ^9^Centre France Chine de la Médecine Chinoise, Selles sur Cher, France; ^10^School of Acupuncture-Moxibustion and Tuina, Beijing University of Chinese Medicine, Beijing, China

**Keywords:** abdominal obesity, meteorological factors, spatial heterogeneity, optimal parameter geographic detector, multiscale geographically weighted regression, China

## Abstract

**Background:**

In recent years, the incidence of abdominal obesity among the middle-aged and older adult population in China has significantly increased. However, the gender disparities in the spatial distribution of abdominal obesity incidence and its relationship with meteorological factors among this demographic in China remain unclear. This gap in knowledge highlights the need for further research to understand these dynamics and inform targeted public health strategies.

**Methods:**

This study utilized data from the 2015 China Health and Retirement Longitudinal Study (CHARLS) to analyze the incidence of abdominal obesity among the middle-aged and older adult population in China. Additionally, meteorological data were collected from the National Meteorological Information Center. Using Moran’s I index and Getis-Ord Gi* statistical methods, the spatial distribution characteristics of abdominal obesity incidence were examined. The influence of various meteorological factors on the incidence of abdominal obesity in middle-aged and older adult males and females was investigated using the q statistic from the Geodetector method. Furthermore, Multi-Scale Geographically Weighted Regression (MGWR) analysis was employed to explore the impact of meteorological factors on the spatial heterogeneity of abdominal obesity incidence from a gender perspective.

**Results:**

The spatial distribution of abdominal obesity among middle-aged and older adult individuals in China exhibits a decreasing trend from northwest to southeast, with notable spatial autocorrelation. Hotspots are concentrated in North and Northeast China, while cold spots are observed in Southwest China. Gender differences have minimal impact on spatial clustering characteristics. Meteorological factors, including temperature, sunlight, precipitation, wind speed, humidity, and atmospheric pressure, influence incidence rates. Notably, temperature and sunlight exert a greater impact on females, while wind speed has a reduced effect. Interactions among various meteorological factors generally demonstrate bivariate enhancement without significant gender disparities. However, gender disparities are evident in the influence of specific meteorological variables such as annual maximum, average, and minimum temperatures, as well as sunlight duration and precipitation, on the spatial heterogeneity of abdominal obesity incidence.

**Conclusion:**

Meteorological factors show a significant association with abdominal obesity prevalence in middle-aged and older adults, with temperature factors playing a prominent role. However, this relationship is influenced by gender differences and spatial heterogeneity. These findings suggest that effective public health policies should be not only gender-sensitive but also locally adapted.

## 1 Introduction

The World Health Organization (WHO) defines obesity as an abnormal or excessive accumulation of fat that may impair health (1). Obesity has become a significant challenge in the global public health arena (2), with its associated complications severely impacting human health (3). Various types of obesity significantly increase the overall mortality rate, particularly abdominal obesity, which is closely linked to numerous health issues and disease risks, including complications from diabetes (4), metabolic syndrome (5), cardiovascular diseases (6), hip fractures (7), lung cancer (8), depression (9), and cognitive impairments (10). The prevalence of obesity has triggered a range of health and economic issues in China, putting immense pressure on the healthcare system (11). Obesity in China is associated with an increased risk of major non-communicable diseases and premature death (12), with abdominal obesity playing a dominant role (13). Abdominal obesity is defined as excessive accumulation of abdominal fat; excessive visceral fat can promote high levels of adipose factors in the portal vein to enter the liver and other body tissues, thereby severely affecting human health (14). Given the aging population structure in China, research on the incidence of abdominal obesity among middle-aged and older adult Chinese is particularly critical.

The etiology of obesity is multifaceted, encompassing biological, genetic, environmental, socio-economic, and psychological behavioral factors (15–18). Current research suggests that the prevalence of obesity is primarily linked to a reduction in physical activity associated with aging, which leads to low energy expenditure, and changes in dietary habits that result in excessive energy intake (19). In recent years, as climate change intensifies and extreme weather events become more frequent, investigating the potential impacts of meteorological changes on human health has emerged as a focal area of interest. Existing studies have indicated a possible correlation between meteorological factors and the incidence of obesity. For instance, one research has shown that temperature, precipitation, and wind speed may indirectly contribute to obesity by reducing outdoor activities in adults (20). Additionally, studies have found that individuals in high-altitude areas tend to lose weight, possibly due to increased metabolic rates and reduced food intake caused by low air pressure and hypoxia (21). Research from South Korea has demonstrated a significant association between ambient temperature and obesity prevalence, potentially linked to increased energy expenditure through cold-induced thermogenesis (22). Similarly, one study in China has discovered that reduced sunlight duration can increase the risk of obesity due to air pollution, potentially due to excessive generation of reactive oxygen species (ROS) and activation of COX-2 (23). Thus, meteorological factors play a crucial role in the development of obesity.

Gender disparities significantly influence the incidence of obesity and its associated health risks. For instance, a study conducted in South Korea revealed significant gender differences in the correlation between abdominal obesity incidence and socio-economic status (24). Another cross-sectional study revealed that cardiac metabolic issues stemming from abdominal obesity differ between genders (25). In terms of various anthropometric indicators of obesity, the mortality rate from cardiovascular diseases tends to be higher in males than females (26). Furthermore, while abdominal obesity is strongly linked to a decline in physical function among males, no significant association is observed in females (27). The research underscores that interventions targeting the prevention or management of abdominal obesity in males may yield more effective outcomes in preventing disability (28). In China, compared to females, males with abdominal obesity have a higher prevalence and risk of metabolic syndrome (29).

Current research is beginning to unravel potential links between meteorological factors, gender disparities, and obesity. Recent evidence suggests that the influence of meteorological factors on obesity may vary among different demographic groups, prompting further investigation into the role of gender differences in environmental inequalities (30). Given the challenges of abdominal obesity in China, as well as the diversity and complexity of its geographical environment, this study employs spatial analysis methods from a gender perspective to explore the relationship between meteorological factors and the incidence of abdominal obesity. We hypothesize that the effects of meteorological factors on the incidence rate of abdominal obesity and its spatial heterogeneity will exhibit significant differences between genders. Utilizing abdominal obesity data from the China Health and Retirement Longitudinal Study (CHARLS) and nationwide meteorological data, this study conducts an in-depth analysis of the relationship between the prevalence of osteoarthritis in middle-aged and older adult Chinese populations and meteorological factors from a gender differences perspective.

## 2 Materials and methods

### 2.1 Materials

#### 2.1.1 Abdominal obesity data

This study utilizes 2015 data on abdominal obesity in middle-aged and older adult individuals from the CHARLS database. The primary aim of the CHARLS database is to collect health data on the Chinese population aged 45 and above to facilitate in-depth research on the aging issue in China. The study’s scope is extensive, covering 28 provinces, 150 cities, and 450 villages. Initiated in 2011, all participants had signed informed consent forms before joining the study. This research has been approved by the Biomedical Ethics Review Committee of Peking University (IRB00001052-11015) and the Human Research Ethics Committee of Newcastle University (H-2015-0290). Detailed information about CHARLS has been published by Zhao et al. (31).

**Figure 1:**
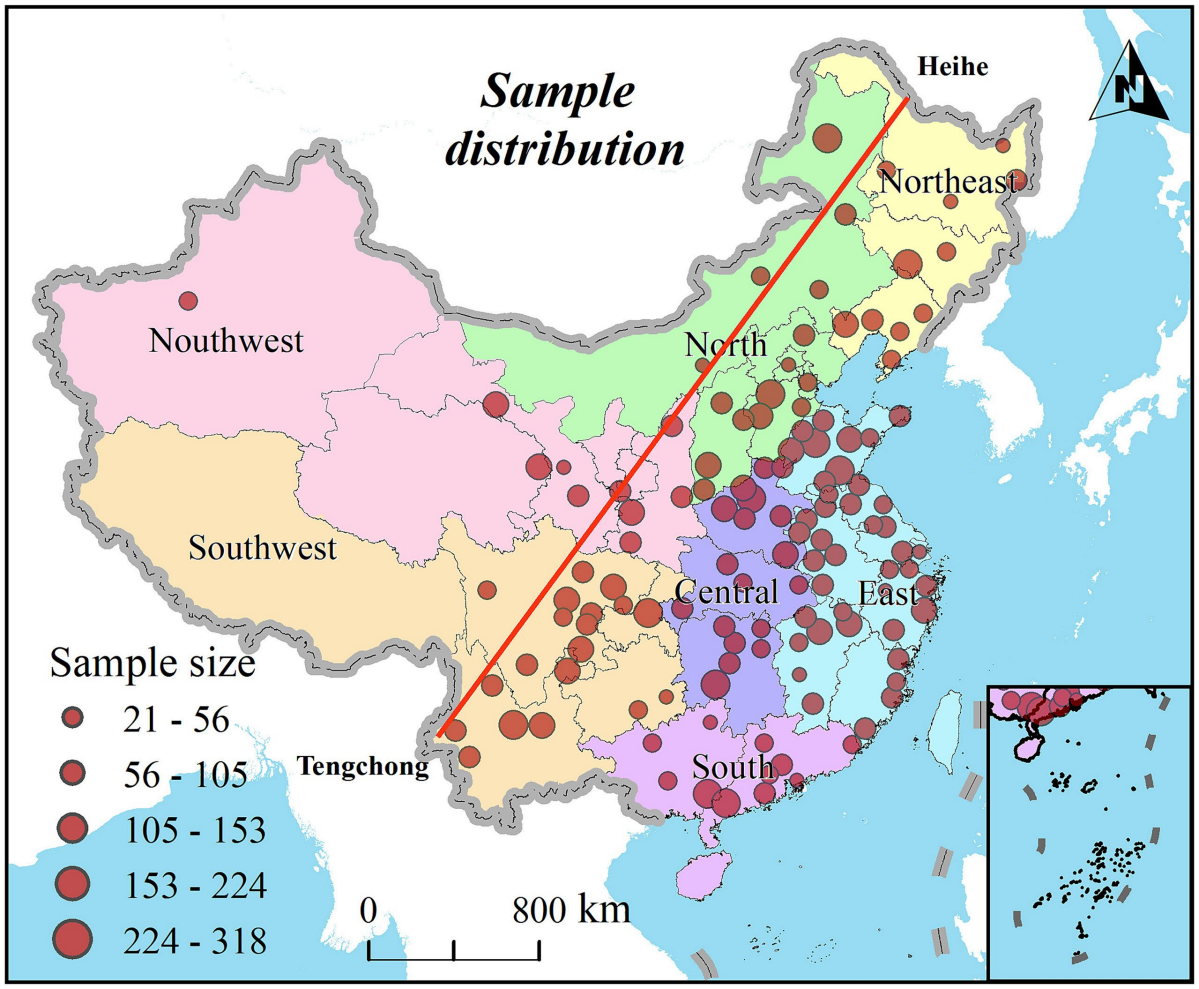
Distribution map of participants.

In accordance with recommendations from the International Diabetes Federation (IDF) tailored for the Chinese populace, abdominal obesity is defined by the Chinese Diabetes Society as a waist circumference of ≥90 cm for males and ≥85 cm for females (12). The prevalence of abdominal obesity in this study was calculated based on the original data collected by the CHARLS. After data preprocessing, which included removing outliers and missing values, a total of 16,249 valid data points were obtained, covering 125 cities in China (shown in Figure 1). The 2015 data on abdominal obesity among middle-aged and older adult individuals were categorized by urban and rural residence, gender, and prefecture-level city regions. The distinction between urban and rural residents was based on their place of residence, either in villages or communities.

Given the geographical, cultural, and economic diversity across China, this study divides the nation into seven geographic regions (32). Figure 1 illustrates the geographical distribution of the participants across 126 survey cities.

#### 2.1.2 Geographical environmental data and inverse distance weighting

The meteorological data were sourced from the National Meteorological Science Data Center, covering 699 meteorological monitoring stations across all 31 provinces and regions. Climate data^1^ from 699 reference and basic weather stations includes precipitation, atmospheric pressure, wind speed, temperature, humidity, and sunshine duration. The IDW (Inverse Distance Weighted) interpolation method was used to convert the climate data of the meteorological stations into grid data with a resolution of 0.1° × 0.1°. The IDW method assumes that the value at observation points closer to the point of prediction is more like it than at further points. IDW interpolation estimates the unknown value of $\hat{Y}$ at the point $x_{0}$ using the values of a given number of observation points $x_{i}$ , weighted by an inverse function of the distance between the unknown point and the observation.

(1) $$\hat{y}\left(x_{0}\right)=\overset{n}{\underset{i=1}{\sum }}W_{i}Y\left(x_{i}\right)$$

Where $W_{i}$ represents the weight function assigned to each observation point $x_{i}$ and $Y\left(x_{i}\right)$ is the measured value at $x_{i}$ . The weights are determined as

(2) $$W_{i}=\frac{d_{i}^{-p}}{\sum_{i=1}^{n}d_{i}^{-p}}$$

Where $d_{i}$ is the Euclidian distance between the predicted point $x_{o}$ and the observation point $x_{i}$ , n is the total number of observation points used in the interpolation of $x_{0}$ , and the exponent p decides how the weight decreases as the distance increases.

Then, the grid data was converted into the raster data. The annual climate data in 2015 based on China’s administrative divisions consists of annual precipitation (AP), annual extreme wind speed (AEWS), annual maximum wind speed (AMWS), annual average wind speed (AAWS), annual minimum humidity (AMH), annual average humidity (AAH), annual minimum temperature (AMIT), annual maximum temperature (AMAT), annual average temperature (AAT), annual sunshine duration (ASD), annual maximum atmospheric pressure (AMAAP), annual minimum atmospheric pressure (AMIAP), and annual average atmospheric pressure (AAAP).

### 2.2 Methods

#### 2.2.1 Moran’s I

Moran’s I index was utilized to quantitatively reveal the spatial clustering characteristics of the prevalence of abdominal obesity among the middle-aged and older adult in China. Moran’s I measure spatial interdependence in data, which characterizes spatial distribution types. The Moran’s I is calculated as:

(3) $$I=\frac{n\sum_{i=1}^{n}\sum_{j=1}^{n}\omega_{ij}\left(y_{i}-\bar{y}\right)\left(y_{j}-\bar{y}\right)}{\left(\sum_{i=1}^{n}\sum_{j=1}^{n}\omega_{ij}\right)\sum_{i=1}^{n}{\left(y_{i}-\bar{y}\right)}^{2}}$$

In Equation (3), I is the global Moran index; n is the total number of regions; i and j denote a region; $\omega_{ij}$ is a matrix of spacial weights which can be defined as the inverse of the distance among locations i and j ; $y_{i}$ and $y_{j}$ is the variable level; $\bar{y}$ is the average variable level. I ranges from −1 to 1. *I* > 0 indicates a positive spatial correlation, and the closer to 1 the higher the spatial agglomeration; *I* < 0 indicates a negative spatial correlation, and the closer to −1 the greater the spatial difference; *I* = 0 indicates spatial random distribution.

Spatial autocorrelation was assessed in this study using the ArcGIS 10.8 platform to compute Moran’s I statistic. Moran’s I values were computed following the methodology proposed by Environmental Systems Research Institute (ESRI) (2022). Initially, the default minimum distance required to estimate spatial autocorrelation was chosen. In cases where this distance was not specified, the spatial autocorrelation tool automatically determined the distance ensuring that each element had at least one neighboring element, with the distance exhibiting the highest Z-score selected as the threshold distance of interest. Subsequently, these threshold distances were utilized in the analysis of Getis-Ord Gi*.

#### 2.2.2 Getis-Ord Gi* analysis

Moran’s I detect spatial clustering features of geospatial data (33). However, it cannot determine whether the clustered values are high or low. Therefore, we used Getis-Ord Gi* analysis to identify high and low-value clusters in the spatial distribution of abdominal obesity prevalence in China. This technology can be implemented in the ArcGIS 10.8 platform. It is calculated as:

(4) $$G_{i}^{*}=\frac{\sum_{j=n}^{n}w_{ij}x_{i}}{\sum_{j=1}^{n}x_{j}}$$

where $G_{i}^{*}$ means the spatial dependency of feature i , and $x_{j}$ is the value of variable X at feature location j . $W_{ij}$ is the spatial weight between features i and j .

#### 2.2.3 Optimal parameter geographic detector

Geodetector is a statistical tool to measure the spatial stratified heterogeneity (SSH) of geography and to reveal the driving forces behind it (34). This method has been widely used in public health and environmental science in recent years. Optimal parameter geographic detector (OPGD) is a development of Geodetector that allows for automatic optimal discretization of data (35). In this study, Geodetector was used to explore the global association of climate factors with morbidity and mortality of stroke in different regions.

(5) $$q=1-\frac{\mathrm{SSW}}{\mathrm{SST}}=1-\frac{\sum_{h=1}^{L}N_{h}\sigma_{h}^{2}}{N\sigma^{2}}$$

(6) $$\lambda =\frac{1}{\sigma^{2}}\left[\overset{L}{\underset{h=1}{\sum }}{\bar{Y}}_{h}^{2}-\frac{1}{N}{\left(\overset{L}{\underset{h=1}{\sum }}\sqrt{N_{h}}\bar{y_{h}}\right)}^{2}\right]$$

In Equation (5), SSW represents the sum of the variances of the response variable in the strata, SST represents the total variance of the response variable for all whole area. N represents the sample units in the whole area and h represents the level of a variable ( h =1, 2, …, L ). $N_{h}$ is the number of units in strata h , $\sigma_{h}^{2}$ and $\sigma^{2}$ are the variance of the response value in strata h and the whole area, respectively. In this study, the discretization scheme with the largest *q*-value was preferred and the range of q-values is (0, 1). The higher the *q*-value converges to 1 indicates that the spatial pattern of the explanatory variable explains more about the response variable. In Equation (6), λ and ${\bar{Y}}_{h}$ represent the mean values of the non-center parameter and the stratum, respectively. In this way, the factor detector can be used to determine whether the q statistic is significant.

The interaction detection module compares the q-values of factors X1 and X2 with the interaction *q*-value (X1∩X2) to quantify the interactions between them, to assess whether the factors are weakened or strengthened by each other or are independent of each other. Typically, the results of interaction detectors can be classified into five types (Table 1). In this study, the “GD” package in R4.1.2 software^2^ was used to implement (a) and (b) functions (35).

**Table 1:** The type of factor interaction expression.

Description	Interaction
q(X1∩X2)<min(q(X1),q(X2))	Weaken, nonlinear
$\min\left(q\left(X1\right),\;q\left(X2\right)\right)<q\left(X1\cap X2\right)<\max\left(q\left(X1\right),\;q\left(X2\right)\right)$	Weaken, unilateral
q(X1∩X2)>max(q(X1),q(X2))	Enhance, bilinear
q(X1∩X2)=q(X1)+q(X2)	Independent
q(X1∩X2)>q(X1)+q(X2)	Enhance, nonlinear

#### 2.2.4 Multiscale geographically weighted regression

Geodetector can only be used to explain the global relationship between meteorological factors and the prevalence of abdominal obesity, ignoring important local differences in the relationship. Multiscale Geographically Weighted Regression (MGWR) is an advanced spatial analysis technique used to uncover local variations and relationships in spatial data (36, 37). As an advancement of Geographically Weighted Regression (GWR), the unique aspect of MGWR is that it allows regression parameters to vary across different spatial scales. This means that MGWR can capture variations and connections at various spatial levels more finely, thus revealing more complex spatial relationships. By modeling the influence of each variable at different spatial scales in detail, MGWR offers researchers more nuanced insights and understanding. Therefore, MGWR considers both the local effects of spatial objects and the scale effects of different spatial processes, enabling a more effective explanation of the spatial heterogeneity in data. The formula for MGWR is as follows:

(7) $$y_{i}=\overset{n}{\underset{i=0}{\sum }}\overset{m}{\underset{j=0}{\sum }}\beta_{bwj}\left(u_{i},v_{i}\right)x_{ij}+\varepsilon_{i}$$

In the formula, $y_{i}$ represents the dependent variable, $x_{ij}$ is the j-th independent variable for observation point i, $\beta_{bwj}$ is the regression coefficient for the j-th dependent variable adjusted for effective bandwidth, $\beta_{bwj}\left(u_{i},v_{i}\right)x_{ij}$ is the regression coefficient of the j-th independent variable at location ( $u_{i}$ , $v_{i}$ ), and $\varepsilon_{i}$ is the random error term. The kernel function and bandwidth selection criteria for the MGWR model are based on the most commonly used quadratic kernel function and the corrected Akaike Information Criterion (AICc), with a smaller AICc indicating a better fit (38). The MGWR 2.2 software developed by Oshan et al. (39) was used in this study to perform the MGWR analyses.

## 3 Results

### 3.1 Study participant characteristics

Table 2 delineates the baseline characteristics of all participants, stratified by gender. The study comprises 16,249 participants from 125 cities across 28 provinces and autonomous regions of China. The data elucidates the distribution characteristics of gender, place of residence, educational level, and waist circumference among middle-aged and older adult Chinese populations. Regarding age, the average age of male participants (60.32 years) is statistically higher than that of females (58.76 years), with a significant difference (*p* < 0.001). In terms of urban–rural classification, most participants reside in rural areas (62.42%), with a slightly higher proportion of males in rural areas compared to females, and a slightly higher proportion of females in urban areas compared to males, though this difference is statistically less significant (*p* = 0.0545). There is a notable gender disparity in educational levels; a significantly higher percentage of females are uneducated compared to males. Among those who have received at least elementary education, the proportion of males is higher than that of females (*p* < 0.001), reflecting gender inequality in education. As for waist circumference, the difference between males and females is not significant (*p* = 0.8250).

**Table 2:** Baseline characteristics of all participants by gender.

Characteristics	Total (*n* = 16,249)	Male (*n* = 7,557)	Female (*n* = 8,692)	*p*
Age, y, mean (SD)	59.49 (10.49)	60.32 (10.48)	58.76 (10.70)	<0.001
Urbanicity, *n* (%)				0.0545
Rural	10,142 (62.42)	4,776 (63.20)	5,366 (61.73)	
Urban	6,107 (37.58)	2,781 (36.80)	3,326 (38.27)	
Education, *n* (%)				<0.001
Uneducated	4,216 (25.96)	901 (11.93)	3,315 (38.15)	
Primary school	7,505 (46.20)	3,883 (51.40)	3,622 (41.68)	
Above primary school	4,522 (27.84)	2,770 (36.67)	1,752 (20.16)	
Waist, cm, mean (SD)	85.46 (13.03)	85.44 (13.08)	85.48 (12.99)	0.8250

Missing data: 6 for education. WC, waist circumference. The mean values and percentages in different quintiles were compared using ANOVA analysis and χ^2^ tests.

### 3.2 The spatial distribution characteristics of abdominal obesity incidence rate

**Figure 2:**
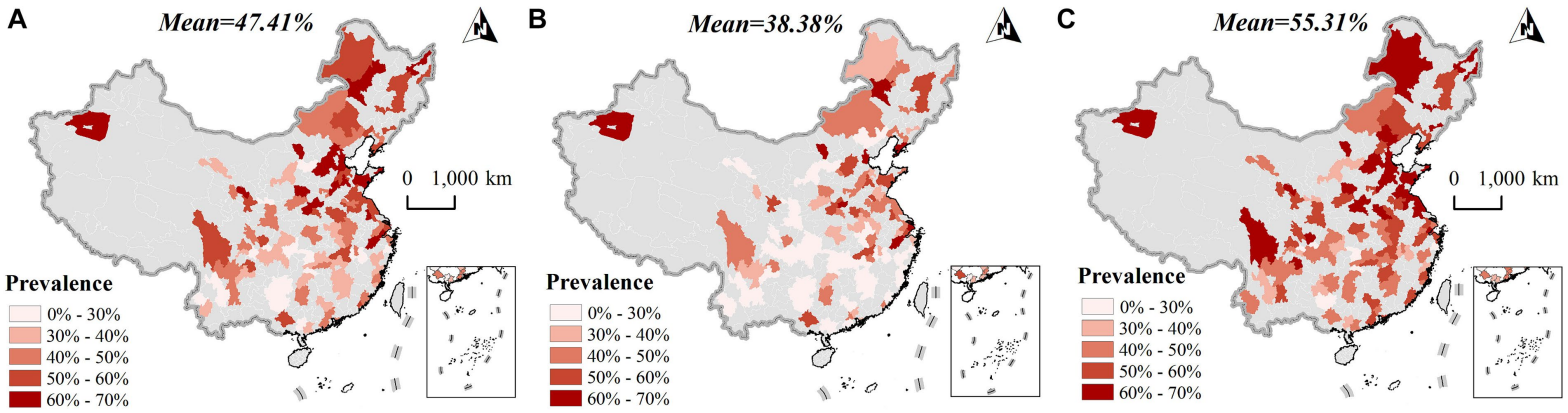
Spatial distribution characteristics of abdominal obesity prevalence in the middle-aged and older adult population in 2015 **(A)** total population; **(B)** male; **(C)** female.

Figure 2A depicts the spatial distribution of abdominal obesity prevalence in 2015 among the total population of middle-aged and older adult individuals in China, while Figures 2B,C illustrate the distribution separately among males and females, respectively. In Figure 2A, the prevalence of abdominal obesity among middle-aged and older adult individuals across China is showcased, with concentrations primarily observed in regions east of the Heihe-Tengchong Line, correlating with areas of higher population density (40). Notably, higher prevalence rates are evident in the northern and central regions, contrasting with lower rates in the southern part of the country. Gender-wise, females exhibit a higher average prevalence of abdominal obesity compared to males, a trend particularly accentuated in southern China. This disparity underscores gender differences in the spatial distribution of abdominal obesity prevalence among middle-aged and older adult individuals in China.

**Figure 3:**
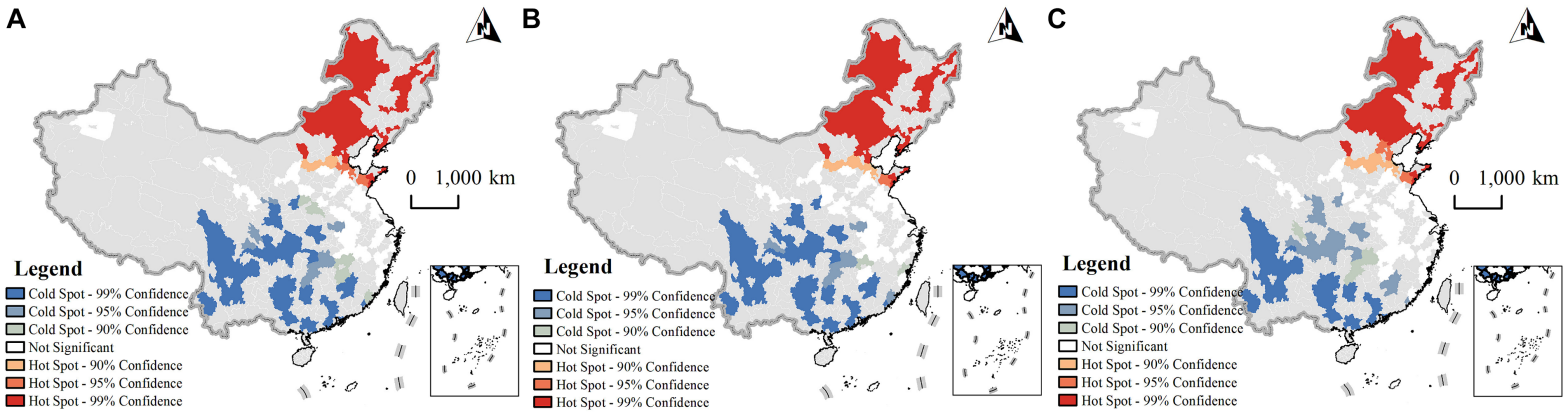
Getis-Ord Gi* hotspot and coldspot analysis of abdominal obesity prevalence in middle-aged and older adult **(A)** total Population; **(B)** male; **(C)** female.

Table 3 presents the results of the spatial autocorrelation analysis of abdominal obesity prevalence among middle-aged and older adult individuals in China. The data unveils a significant spatial clustering of abdominal obesity prevalence across the total population, as well as within male and female subgroups. Additionally, Figure 3 delineates the spatial distribution of hotspots and coldspots for abdominal obesity prevalence among this age group across China. On an overall scale, hotspots for abdominal obesity prevalence are predominantly concentrated in the Northeast region of China, while coldspots primarily reside in the Southwest region, indicating a distinct spatial division. From a gender standpoint, the spatial distribution of hotspots and coldspots for abdominal obesity prevalence in males and females closely mirrors that of the total population, suggesting that gender may not significantly differentiate the spatial aggregation of abdominal obesity.

**Table 3:** Spatial autocorrelation of abdominal obesity incidence rate in middle-aged and older adult Chinese.

	Total	Male	Female
Moran’s I	0.206	0.170	0.179
Z-score	13.677	11.384	11.987
*p*-value	<0.001	<0.001	<0.001

### 3.3 Analysis of factors influencing abdominal obesity prevalence in middle-aged and older adult

#### 3.3.1 Global relationships between meteorological factors and the abdominal obesity prevalence

Table 4 reports the overall impact of meteorological factors on the prevalence of abdominal obesity in the middle-aged and older adult population based on factor detector. All indicators except AMIAP passed the test of significance (*p* < 0.01). Looking at the total population, the impact of the AMAT with a *q*-value of 0.361 was the most significant, followed by the AAT with a *q*-value of 0.327, AP with a *q*-value of 0.308, ASD with a *q*-value of 0.288, AAH with a *q*-value of 0.252, and AAWS with a *q*-value of 0.244. This suggests that temperature indicators are the dominant meteorological factor influencing the incidence of abdominal obesity. Low-temperature environments may contribute to obesity by influencing various aspects of the body’s energy intake, fat synthesis and metabolism, food digestion and absorption, and lifestyle habits. However, atmospheric pressure and wind speed appear to have minimal effect, suggesting they are not directly associated with the physiological mechanisms of obesity. Regarding the male population, the explanatory power of meteorological indices on the prevalence of abdominal obesity decreased overall. The order of significance was as follows: AMAT with a *q*-value of 0.299, followed by AAT with a *q*-value of 0.277, AP with a *q*-value of 0.251, AMIT with a *q*-value of 0.237, ASD with a *q*-value of 0.227, and AAWS with a *q*-value of 0.218. For the female population, AAT with a *q*-value of 0.358 had the most significant impact, followed by AMAT with a *q*-value of 0.348, ASD with a *q*-value of 0.316, AMIT with a *q*-value of 0.298, AP with a *q*-value of 0.278, and AAH with a *q*-value of 0.257. The prevalence of abdominal obesity is therefore less affected by meteorological factors in men than in women. This may be due to the fact that the basal metabolic rate of women is usually lower than that of men, which means that women are more susceptible to meteorological factors, which in turn affects the incidence of obesity (41).

**Table 4:** Factor detector results.

Factors	Total		Male		Female	
	*q*-value	*p*	*q*-value	*p*	*q*-value	*p*
AP (mm)	0.308	<0.001	0.251	<0.001	0.278	<0.001
AEWS (m/S)	0.093	0.004	0.082	0.005	0.074	0.013
AMWS (m/s)	0.141	0.001	0.122	0.001	0.125	0.002
AAWS (m/s)	0.244	<0.001	0.218	<0.001	0.142	<0.001
AMH (%)	0.168	<0.001	0.139	<0.001	0.157	<0.001
AAH (%)	0.252	<0.001	0.217	<0.001	0.257	<0.001
AMIT (°C)	0.272	<0.001	0.237	<0.001	0.298	<0.001
AMAT (°C)	0.361	<0.001	0.299	<0.001	0.348	<0.001
AAT (°C)	0.327	<0.001	0.277	<0.001	0.358	<0.001
ASD (hours)	0.288	<0.001	0.227	<0.001	0.316	<0.001
AMAAP (hpa)	0.139	<0.001	0.156	0.001	0.118	0.001
AMIAP (hpa)	0.083	0.070	0.063	0.117	0.073	0.077
AAAP (hps)	0.086	0.021	0.080	0.028	0.073	0.038

AP, annual precipitation; AEWS, annual extreme wind speed; AMWS, annual maximum wind speed; AAWS, annual average wind speed; AMH, annual minimum humidity; AAH, annual average humidity; AMIT, annual minimum temperature; AMAT, annual maximum temperature; AAT, annual average temperature; ASD, annual sunshine duration; AMAAP, annual maximum atmospheric pressure; AMIAP, annual minimum atmospheric pressure; AAAP, annual average atmospheric pressure.

Overall, temperature, precipitation, sunshine, humidity, wind speed, and atmospheric pressure all influence the prevalence of abdominal obesity. Notably, the effects of temperature and sunshine duration on abdominal obesity prevalence in women are more pronounced than in men.

#### 3.3.2 Interaction effect of meteorological factors

**Figure 4:**
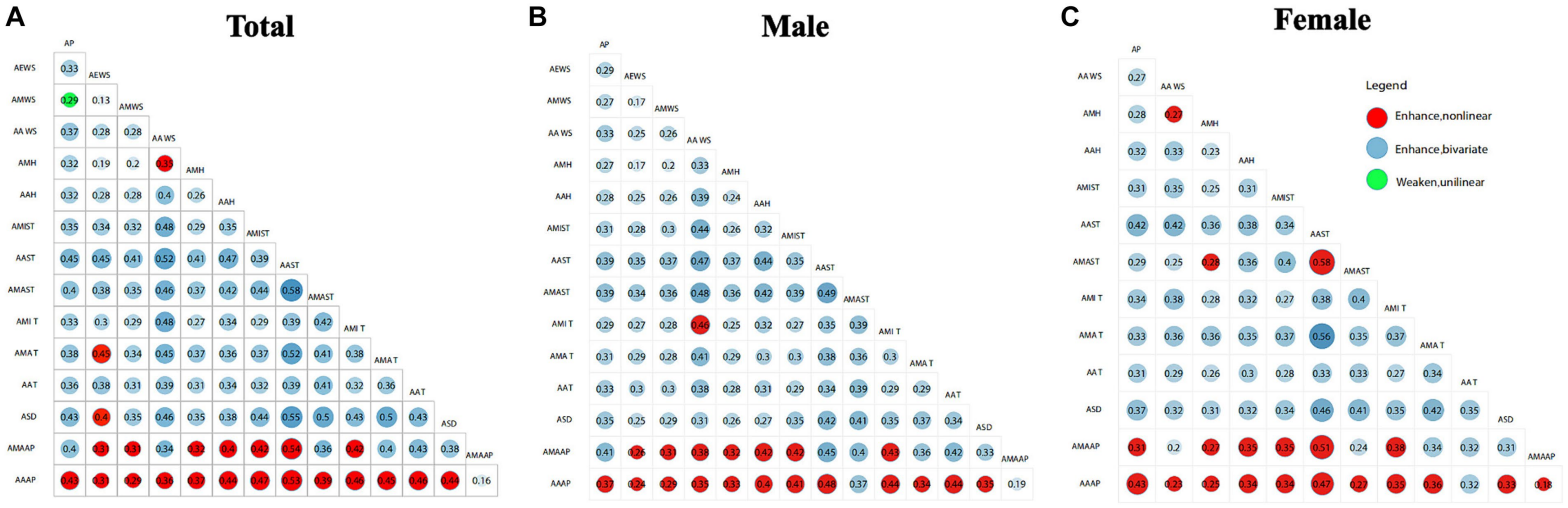
Interaction analysis results **(A)** total Population; **(B)** male; **(C)** female. AP, annual precipitation; AEWS, annual extreme wind speed; AMWS, annual maximum wind speed; AAWS, annual average wind speed; AMH, annual minimum humidity; AAH, annual average humidity; AMIT, annual minimum temperature; AMAT, annual maximum temperature; AAT, annual average temperature; ASD, annual sunshine duration; AMAAP, annual maximum atmospheric pressure; AMIAP, annual minimum atmospheric pressure; AAAP, annual average atmospheric pressure.

Figure 4 presents the results of interaction analysis for meteorological factors that significantly affect the prevalence of abdominal obesity in the middle-aged and older adult population. The interactions between most meteorological factors demonstrate bivariate enhancement, indicating that the combined effects of two meteorological factors together are greater than their individual effects (41).

From the perspective of the total population, the AAAP shows strong nonlinear enhancement with the AMIT at 0.461, AAT at 0.459, and AMAT at 0.445. For the male population, AAAP shows strong nonlinear enhancement with the annual minimum temperature AMIT at 0.440, annual average temperature AAT at 0.44, and annual average humidity AAH at 0.40. From the female population perspective, AAAP shows strong nonlinear enhancement with AP at 0.425, AMAT at 0.364, and AMIT at 0.354. This reflects a phenomenon in which an otherwise insignificant atmospheric pressure factor has a significantly increased effect on abdominal obesity when combined with other factors due to an interaction effect. Overall, the annual average atmospheric pressure exhibits nonlinear enhancement in interaction with temperature, humidity, and precipitation across different meteorological factors, yet these interactions do not exhibit significant gender disparities.

#### 3.3.3 Local relationships between major meteorological factors and the abdominal obesity prevalence

To reveal the impact of meteorological factors on the spatial heterogeneity of abdominal obesity among middle-aged and older adult individuals, this study employed the MGWR method, providing a comprehensive analysis of how meteorological factors influence the spatial variations in abdominal obesity prevalence. The study selected the top six meteorological factors based on their q-values and conducted detailed analyses from the perspectives of the total population, males, and females. The variance inflation factor (VIF) of all six indicators is less than 7, indicating that there is no multicollinearity between the indicators. In the regression analysis results, the higher the *R*^2^ value and Log-likelihood, the smaller the AICc and RSS values, indicating a better and more accurate model fit. From the data in Table 5, it can be inferred that the MGWR model outperforms the OLS model for all model parameters. Therefore, MGWR is more accurate than the OLS model and can be used to analyze the relationship between meteorological factors and the incidence of abdominal obesity more precisely.

**Table 5:** Comparison of global OLS models and MGWR models.

Model parameters	Total		Male		Female	
	OLS	MGWR	OLS	MGWR	OLS	MGWR
Residual sum of squares (RSS)	62.695	62.695	85.739	62.695	84.591	65.909
Log-likelihood	−148.371	−134.24	−153.805	−148.371	−152.962	−137.365
AICc	313.983	301.484	324.850	313.983	323.165	308.055
*R*^2^	0.371	0.498	0.314	0.371	0.323	0.473
Adjust *R*^2^	0.339	0.437	0.279	0.339	0.289	0.408

**Figure 5:**
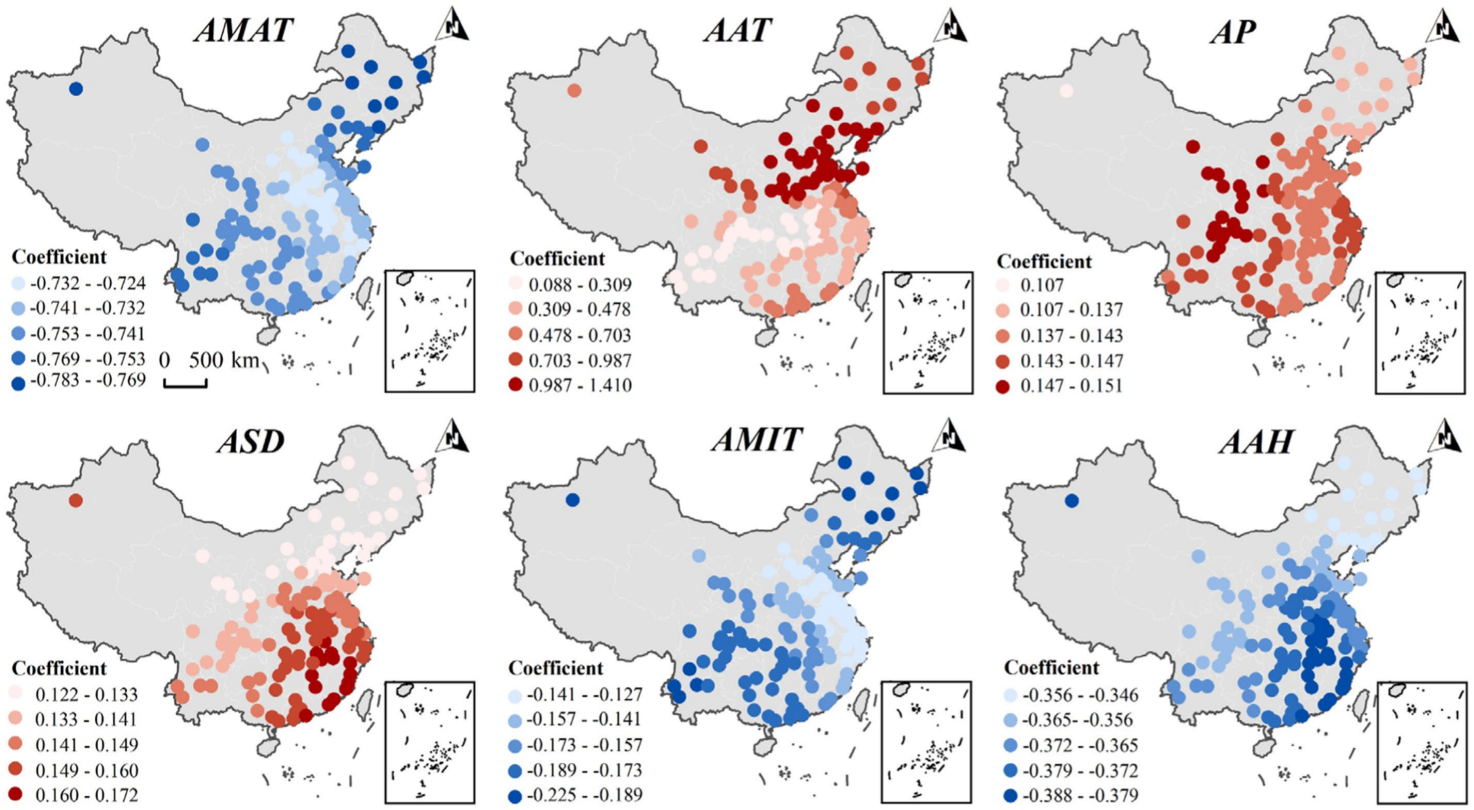
Spatial heterogeneity of the impact of key meteorological factors on the incidence of abdominal obesity in the overall population of the older adult in 2015. The legend represents the interval of MGWR local regression coefficient values; red indicates a positive correlation, and blue indicates a negative correlation in the figure.

Figure 5 illustrates the spatial variation in the effect of major meteorological factors on the prevalence of abdominal obesity in the total population. The regression coefficients for AMAT and AMIT ranged from −0.732 to −0.769 and from −0.141 to −0.189, respectively, and both showed a spatial pattern of decreasing from the northeast and south-west toward the center. The regression coefficients for AAH ranged from −0.356 to −0.379 and were the highest in the south-central region. In contrast, AAT, AP, and ASD were positively associated with the prevalence of abdominal obesity in all regions, with regression coefficients ranging from 0.088 to 1.410 for AAT and showing a spatial pattern of decreasing from north-east to south-west, and 0.122 to 0.172 for ASD, and a spatial pattern of decreasing from north to south. The regression coefficients for AP ranged from 0.107 to 0.151, and the effect was strongest in the west and weakest in the northeast. The regression coefficients of AP ranged from 0.107 to 0.151, showing a spatial pattern of high in the west and low in the northeast. These findings imply that the association between meteorological factors and the prevalence of abdominal obesity is influenced by geographic location, highlighting the importance of local geographic context.

**Figure 6:**
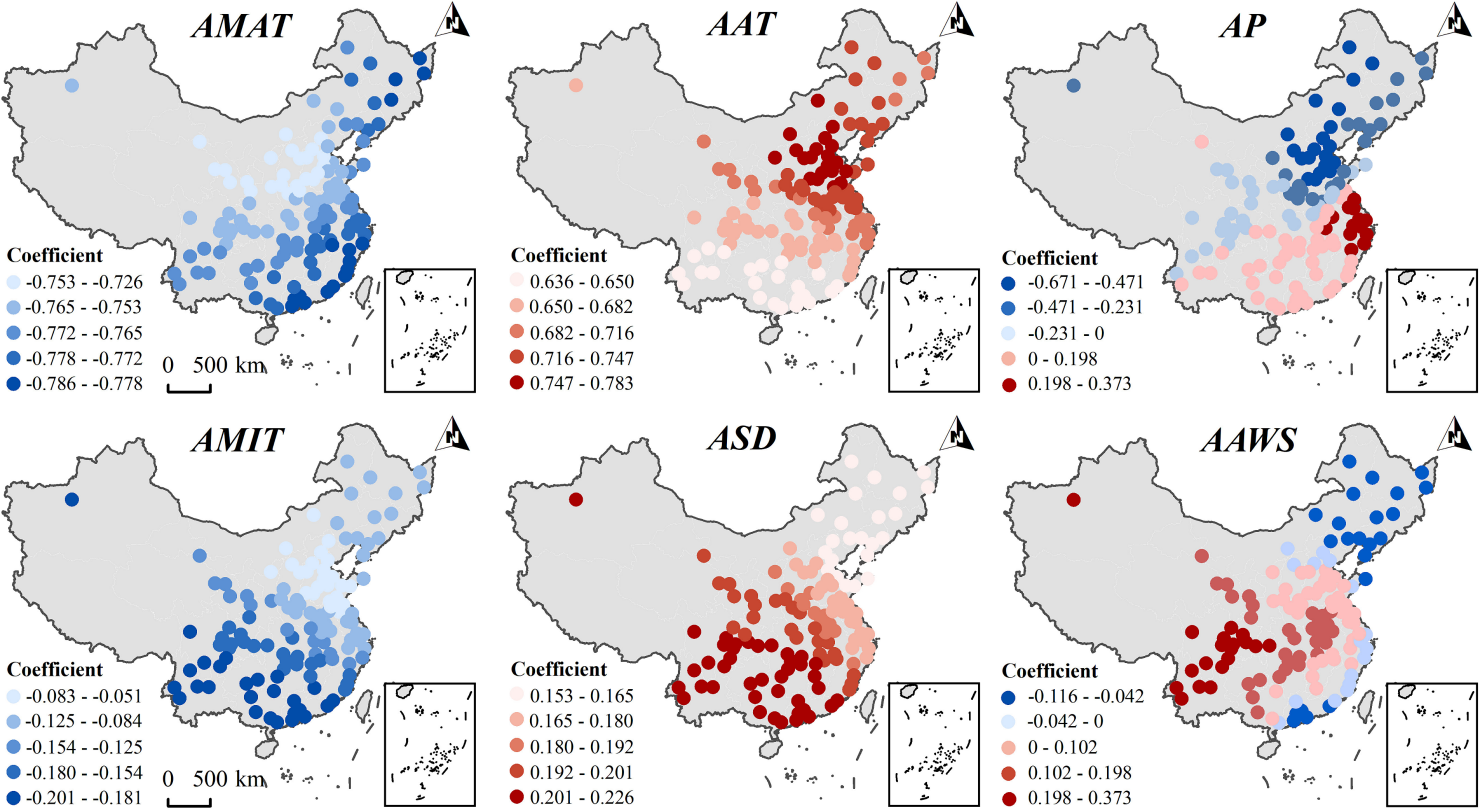
Spatial heterogeneity of the impact of key meteorological factors on the incidence of abdominal obesity in the male population of the older adult in 2015.

By gender, Figure 6 illustrates the spatial variation in the effect of major meteorological factors on the prevalence of abdominal obesity in males. The AMAT and the AMIT show a negative correlation with the incidence of abdominal obesity across the entire country. Conversely, the AAT and the ASD exhibit a positive correlation with the incidence of abdominal obesity nationwide. Interestingly, there were significant spatial differences in the correlation between AP and abdominal obesity in men: a negative correlation in the northern region and a positive correlation in the southwestern, southern, and eastern regions of China, and the regression coefficients differed by a maximum of 1.044 between cities. In addition, AAWS was negatively associated with the prevalence of abdominal obesity in Northeast China, with regression coefficients ranging from 0 to 0.116; however, it was positively associated with Southwest, Northwest, and Central China, with regression coefficients ranging from 0 to 0.373. This implies that the relationship between meteorological factors and the prevalence of abdominal obesity may change in diametrically opposite ways due to geographical differences.

**Figure 7:**
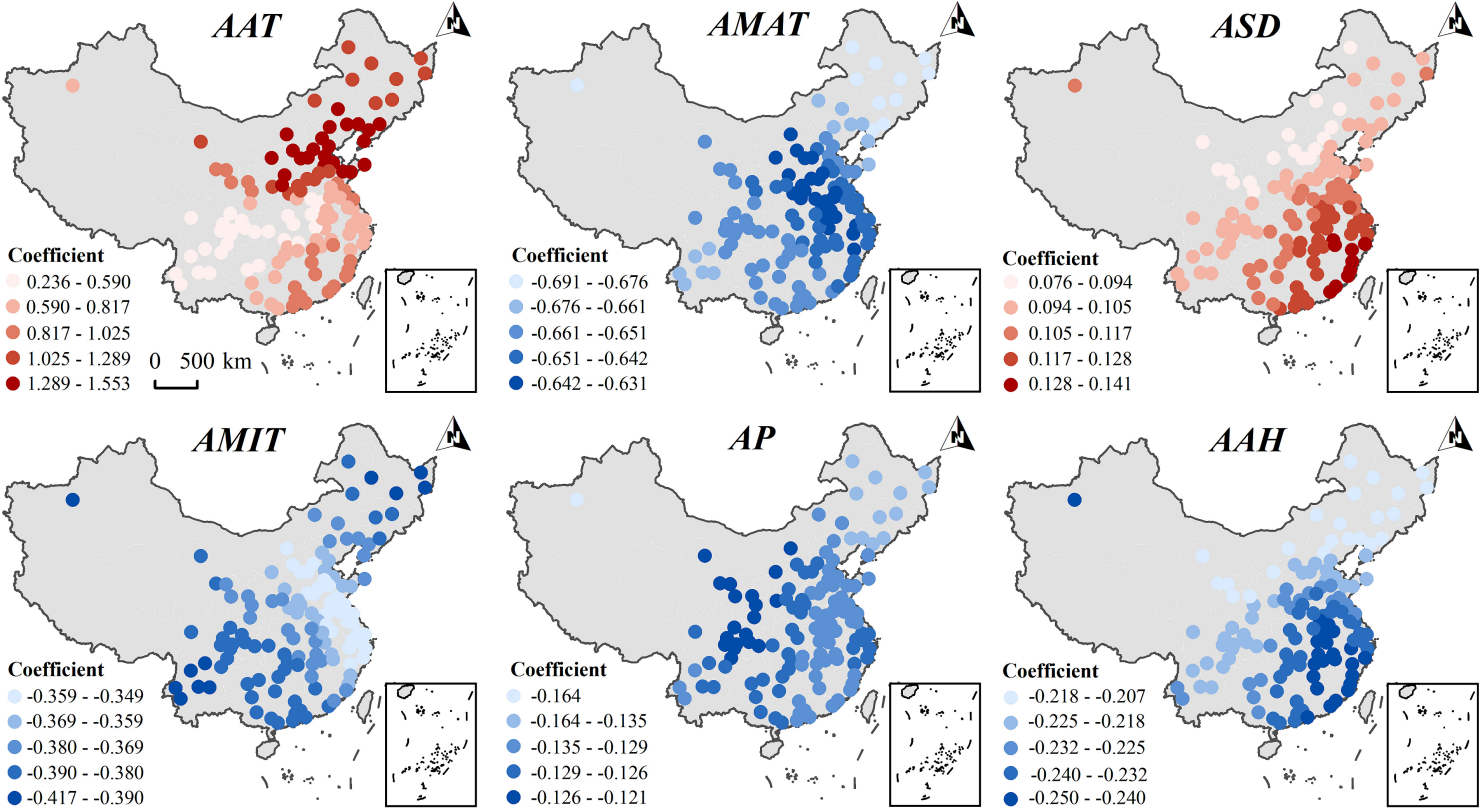
Spatial heterogeneity of the impact of key meteorological factors on the incidence of abdominal obesity in the female population in 2015.

Figure 7 shows the spatial variation in the effect of major meteorological factors on the prevalence of abdominal obesity in females and generally shows a similar spatial pattern to that of the total population. The AAT and ASD exhibit a positive correlation with the incidence of abdominal obesity across the entire country. In contrast, the AMAT, AMIT, AP, and AAH show a negative correlation with the incidence of abdominal obesity nationwide.

Comparative analysis shows that the influence of the annual maximum temperature (AMAT) on the incidence of male abdominal obesity is more pronounced in the Northeast, East, and South China regions, while for females, it is stronger in North, Central, and East China. The impact of the AAT on male abdominal obesity is weaker in South China, and for females, it is less pronounced in Southwest China. AP negatively affects the incidence of male abdominal obesity in the Northeast and North China, but positively in East and South China. In contrast, for females, it has a negative impact across all regions, with a stronger negative effect in the Northwest and Southwest. The influence of AMIT on the incidence of male abdominal obesity is weaker in Northeast China, whereas for females, the effect is stronger in the same region. ASD more significantly impacts the incidence of male abdominal obesity in the Southwest and Northwest, while for females, its impact is stronger in East China. In conclusion, there were gender differences in the spatial heterogeneity of the effects of AMAT, AAT, AMIT, AP, and ASD on the prevalence of abdominal obesity.

## 4 Discussion

This study, from a gender perspective, examines the correlation between the spatial distribution characteristics of abdominal obesity incidence among middle-aged and older adult individuals in China and meteorological factors. It reveals a decreasing spatial distribution trend of abdominal obesity incidence from northwest to southeast, accompanied by significant positive spatial autocorrelation. Hotspots of abdominal obesity incidence are concentrated in North and Northeast China, while cold spots primarily exist in Southwest China, indicating a clear spatial division between areas with high and low incidence rates. Additionally, the study identifies a complex relationship between meteorological factors and abdominal obesity prevalence using global and local models. Differences in spatial location and gender play a crucial role in this relationship, highlighting the sensitivity of gender-specific physiological responses to meteorological conditions and the impact of geographic disparities on abdominal obesity pathogenesis. Thus, considering both gender and spatial location is essential when assessing the relationship between meteorological factors and abdominal obesity risk.

### 4.1 Effect of meteorological factors on abdominal obesity incidence from a geographical perspective

Studies on the spatial distribution patterns of abdominal obesity prevalence and spatial heterogeneity in the effects of meteorological factors will provide important insights for the development of effective and targeted public health strategies. In this study, the spatial distribution pattern of abdominal obesity prevalence, the dominant meteorological factors affecting the prevalence of abdominal obesity, and their spatial heterogeneity were revealed based on four important geospatial analysis techniques, namely, Moran’s I, Getis-Ord Gi* analysis, Geodetector, and the MGWR model, respectively.

Firstly, the incidence of abdominal obesity in the middle-aged and older adult population in China shows a decreasing spatial distribution from northeast to southwest, with significant spatial autocorrelation. The hot spots of abdominal obesity are mainly concentrated in North China and Northeast China, while the cold spots are mainly distributed in Southwest China, which presents an obvious spatial division of high and low-incidence areas. And there is a difference of more than 60% between the cities with the largest and the smallest prevalence rates, which is a huge geographical difference implying that the obesity problem in the northern region of China is more in need of attention from public health policies.

Secondly, this study comprehensively analyzed the association between abdominal obesity prevalence and meteorological factors based on the Geodetector and MGWR model, which helps to understand the reasons for the significant spatial variations in abdominal obesity prevalence. It was found that temperature, precipitation, and humidity had significant effects on the incidence of abdominal obesity, and there was spatial heterogeneity in the intensity of the effects of the factors. In terms of temperature factor, the body uses more energy to maintain body temperature in colder environments, but it also prompts an increased intake of high-calorie foods for energy (42). If the energy intake exceeds the body’s energy needs, it can lead to the accumulation of abdominal fat (43). It has also been shown that temperature has an impact on gut flora (44), with an increase in the number of Firmicutes in colder environments (45), leading to an increase in the risk of obesity by facilitating the body’s efficient absorption of more energy from food (46, 47). It is worth noting that the Northeast and Southwest regions are the coldest and hottest regions in China, respectively, and their extremes of temperature have a more pronounced effect on obesity prevalence, which explains the stronger effect of AMIT and AMAT on obesity prevalence in this region. In terms of humidity, the body needs to expend more energy to maintain body temperature and metabolic regulation in a higher humidity environment (48), which may help to burn excess fat and thus reduce the risk of obesity (49). Meanwhile, the Southeast region has the highest average annual humidity in China, which may lead to a stronger link between humidity factors and the risk of obesity prevalence in this region compared to other regions. In terms of precipitation, on the one hand, rainy weather reduces the time of sunlight exposure, which affects the body’s absorption of vitamin D, leading to metabolic disorders (50), and ultimately affecting the decomposition and utilization of fat (50); on the other hand, rainy weather affects the time of outdoor activities to a certain extent, which means that people consume less energy due to exercise (20), which may lead to an imbalance between energy intake and consumption, and an increase in the risk of obesity (51). As precipitation is very scarce in Northwest China, this may lead to a greater sensitivity to precipitation-induced obesity, which in turn leads to a higher correlation between the two.

Finally, it is worth noting that the spatial variation in the prevalence of abdominal obesity is the result of a complex interaction of different elements (37, 38). According to interaction detection analyses, the otherwise insignificant factor of barometric pressure significantly increases its effect on abdominal obesity when combined with other factors. For example, in environments with lower barometric pressure and higher humidity, the body needs to expend more energy to maintain body temperature and regulate metabolism, which may help burn excess fat and reduce the risk of obesity. In addition, there may be limitations in considering only meteorological indicators. Relevant studies have shown that socio-economic factors, lifestyle, dietary habits, and healthcare resource allocation may also have direct or indirect effects. For example, dietary habits in northern China favor manufactured wheat products with high carbohydrate intake (52, 53). In contrast, dietary habits in the southern region tend to be more varied (54, 55), so differential dietary habits may influence the spatial pattern of the incidence of abdominal obesity. Overall, these findings provide important information and guidance for developing more precise and effective public health strategies targeting abdominal obesity among the middle-aged and older adult populations in China.

### 4.2 Potential mechanisms for the formation of gender differences

Our study confirms previous findings that meteorology has a significant impact on obesity prevalence (22, 56, 57) but also provides a further novel finding that women may be more susceptible to meteorological factors than men and that there is spatial heterogeneity, particularly in temperature and sunlight. This may be associated with several mechanisms. Prolonged residence in extremely cold or hot regions may increase the risk of severe depression (58). Inflammation plays a critical role in the pathogenesis of depression (59), and females may be more susceptible to the emotional and behavioral changes induced by inflammation compared to males (60). Studies suggest a correlation between obesity in females and severe depression, as obesity and depression often co-occur (61). Temperature variations may more readily induce changes in emotional and behavioral patterns in females, leading to the onset of abdominal obesity (62). Cold temperatures can activate brown adipose tissue (BAT) through the sympathetic nervous system (62). BAT plays an important role in regulating body weight and is more abundant and functionally active in females than in males (63). The amount of BAT inversely correlates with body mass index and plays a potential role in adult metabolism (63), typically activated during cold exposure to increase energy expenditure through adaptive thermogenesis (64). The activation of BAT by temperature changes, and its role in regulating body weight, may be more pronounced in women.

Sunlight serves as the primary source of vitamin D synthesis in the skin (65). Experimental research has shown that obesity is associated with low levels of vitamin D, which is linked to the differentiation and growth of adipose tissue. Obesity can result from altered gene expression or the regulation of parathyroid hormone, calcium, and leptin levels due to vitamin D deficiency (66). Females, due to differences in lifestyle, may receive less sunlight exposure, leading to lower vitamin D levels. Consequently, changes in sunlight exposure may have a more sensitive impact on the incidence of abdominal obesity in females (67). Variations in sunlight exposure correlate significantly with Seasonal Affective Disorder (SAD) symptoms and heightened depression risk (68). Given females’ heightened susceptibility to emotional fluctuations, sunlight exposure might mitigate emotional eating and enhance physical activity engagement by improving psychological well-being, potentially influencing abdominal obesity onset (69).

### 4.3 Implications for public health policy

The findings of this study offer valuable insights for public health policy, particularly regarding the prevention and intervention of abdominal obesity among middle-aged and older adult individuals in China. The geographic variation in abdominal obesity prevalence calls for region-specific public health strategies, especially in regions with high incidence rates, such as North and Northeast China. These strategies may entail improving dietary habits, enhancing access to public health facilities, promoting health education, and ensuring equitable distribution of medical resources. The study emphasizes significant gender disparities in the influence of temperature and sunlight on abdominal obesity incidence and spatial distribution. This highlights the necessity of considering gender-specific factors in health interventions. Public health policies should prioritize women’s health guidance, including promoting adequate sunlight exposure for optimal vitamin D levels and offering strategies to prevent vitamin D deficiency during winter months. Furthermore, recognizing the interconnectedness of temperature, sunlight, depression, and brown adipose tissue, efforts to strengthen seasonal psychological health support and activate brown adipose tissue function are essential in reducing abdominal obesity incidence among women. Given the complex interactions between meteorological factors, public health policies should consider the multifaceted impacts of environmental factors. Developing interdisciplinary, comprehensive health interventions tailored to diverse climatic conditions and gender-specific requirements is imperative.

Although individual meteorological factors may affect abdominal obesity incidence differently across genders, their interaction predominantly enhances effects in a bivariate manner, without clear gender distinctions. This bivariate enhancement indicates that the impact of a single meteorological factor on abdominal obesity cannot be considered in isolation; rather, it results from combined interactions. Significantly, these interactions do not display substantial gender differences, suggesting fundamental physiological or environmental mechanisms universally applicable across genders. This insight emphasizes the importance of not only considering individual meteorological factors but also comprehensively assessing their complex interactions when designing interventions to reduce abdominal obesity risk. While gender considerations are vital in many health aspects, addressing meteorological factor interactions with abdominal obesity incidence may require greater focus on their commonalities and universal impacts across genders.

### 4.4 Significance and limitations

Existing research has already demonstrated a correlation between meteorological factors, such as temperature and sunlight, and the incidence of obesity (22, 23). This study corroborates these findings by highlighting the significant impact of meteorological factors on the incidence of abdominal obesity, aligning with the conclusions of existing literature. Uniquely, this research is the first to examine the influence of meteorological factors on the incidence of abdominal obesity among the middle-aged and older adult population in China from a gender perspective. It reveals gender differences in the impact of meteorological factors on the incidence and spatial heterogeneity of abdominal obesity, contrasting with previous studies that focused solely on the relationship between obesity and single meteorological factors or from a gender perspective alone (25, 29).

Additionally, this study employs innovative research methodologies to uncover novel insights. Previous research primarily concentrated on the correlation between obesity and individual meteorological factors, often overlooking their interactions and potential geographic influences. Utilizing diverse spatial analysis techniques, this research delves into the spatial distribution characteristics of abdominal obesity incidence across various Chinese regions and examines the interactions between meteorological factors. It elucidates the connection between the spatial distribution patterns of abdominal obesity and meteorological factors, providing a fresh perspective on the impact of geographic and environmental factors on obesity.

While this study offers new insights into the spatial distribution of abdominal obesity among the middle-aged and older adult population in China and its relationship with meteorological factors, it also has limitations. A major constraint is that the data is derived from a cross-sectional survey conducted in 2015, which limits the ability to analyze trends in abdominal obesity over time. Although CHARLES is a national survey, the sample does not fully cover the population in all regions of China, which makes it difficult to establish a causal relationship between meteorological factors and the prevalence of abdominal obesity. Future research could explore the dynamic relationship between abdominal obesity and meteorological factors more comprehensively through long-term longitudinal studies combined with local health statistics. In addition, this study only examined the spatial patterns affecting the prevalence of abdominal obesity from a meteorological perspective. Future studies should still consider a wider range of geographic and socio-economic factors (70), as well as other environmental influences, including air pollution and urbanization (71, 72), which could help to further explore the complex mechanisms of abdominal obesity. Meanwhile, this study only explored the factors affecting the prevalence of obesity in middle-aged and older adult people, and it is equally important to carry out a wide range of investigations at different ages in the future (73, 74). Given the importance of gender differences, further research should also delve into how gender affects the mechanisms of abdominal obesity and develop more effective prevention and intervention measures tailored to different gender groups. These studies will further enrich our understanding of the epidemiological characteristics and determinants of abdominal obesity, providing more comprehensive guidance for public health practice.

## 5 Conclusion

This study explored the spatial distribution pattern of abdominal obesity prevalence and its association with meteorological factors among middle-aged and older adult individuals in China. It revealed significant gender differences and spatial heterogeneity in the effects of these factors on abdominal obesity prevalence. These findings offer valuable insights for public health policy, providing a fresh perspective on abdominal obesity spatial distribution within this demographic. They also lay a scientific groundwork for crafting more effective prevention and intervention strategies that account for gender disparities. Future research should delve deeper into the mechanisms underlying the differential impact of meteorological factors on abdominal obesity incidence in males and females. Such insights will facilitate the development of targeted approaches to address these nuanced influences, potentially enhancing the effectiveness of public health strategies and interventions.

## Data availability statement

The original contributions presented in the study are included in the article/supplementary material, further inquiries can be directed to the corresponding authors.

## Author contributions

CY: Writing – original draft. JY: Writing – original draft. JW: Writing – original draft. TW: Writing – original draft. HL: Writing – review & editing. YW: Writing – review & editing. HW: Writing – review & editing. SF: Writing – review & editing. YL: Writing – original draft.

## Footnotes

^1^http://data.cma.cn/data/

^2^https://mirror.lzu.edu.cn/CRAN/
